# The Immediate and Lasting Effects of Resident Summer Camp on Movement Behaviors Among Children

**DOI:** 10.3389/fped.2022.912221

**Published:** 2022-06-28

**Authors:** Tetsuhiro Kidokoro, Yuji Minatoya, Natsuko Imai, Akiko Shikano, Shingo Noi

**Affiliations:** ^1^Research Institute for Health and Sport Science, Nippon Sport Science University, Tokyo, Japan; ^2^Graduate School of Health and Sport Science, Nippon Sport Science University, Tokyo, Japan

**Keywords:** physical activity, sedentary behavior, sleep, youth, summer vacation

## Abstract

This study aims to examine the immediate and lasting effects of resident summer camp on movement behaviors among children with repeated pre-, during-, and post-intervention measurements. In total, 21 children (aged 10.3 ± 1.2 years, 17 boys and 4 girls) participated in a 31-day nature-based resident summer camp in Japan. Daily children's movement behaviors (moderate-to-vigorous physical activity (MVPA), sedentary behavior (SB), and sleep) were continuously monitored before, during, and after the summer camp (i.e., 75 continuous days). It was found that the children engaged more time in MVPA (9.6%), less time in SB (58.0%), had more steps (22,405 steps/day), and an earlier midpoint of sleep (0:24 a.m.) in the summer camp as compared to the other periods (before and after the camp). However, the children engaged in unfavorable behaviors [reduction in MVPA (3.6%), increased SB (67.3%), and a later midpoint of sleep (1:32 a.m.)] during the summer vacation after the camp. This study indicates that the resident summer camp was effective in improving children's movement behaviors during the camp. However, the lasting effects were negligible or at least limited after its completion.

## Introduction

Summer vacation is a critical period for healthy development among children. Indeed, emerging evidence suggests that the summer vacation is a period of gaining excess body weight (1–4) and reduced cardiorespiratory fitness (CRF) (5, 6) compared with a school term. These unfavorable changes in body weight and CRF during the summer vacation can be explained by the “Structured Days Hypothesis (SDH)” (7). It posits that the presence of a structured environment, defined as pre-planned, segmented, and adult-supervised compulsory environment, is crucial for children to engage in preferable movement behaviors [i.e., increase in physical activity (PA), reduction in sedentary behaviors (SB), and earlier bedtime] (7). According to the SDH, children engage in less preferable behaviors during the summer vacation (typically less-structured) as compared to a school term (more-structured environment provided by the school). While few studies have examined children's movement behaviors during the summer vacation in contrast to the school term, evidence showed that children spent significantly less time in PA (8–11), more time in SB (10–12) and recreational screen time (10, 12), and their sleep time shifted to later (9) during the summer vacation. As children's movement behaviors are significantly associated with cardiometabolic and psychological health (13, 14), it becomes necessary to promote healthy behaviors during summer vacation.

One potential solution is to provide a structured program during the summer vacation, such as a summer camp. While limited evidence is available regarding the impact of summer camps on children's movement behavior, preliminary findings suggest that participation in summer camps are effective in improving PA, SB, and sleep among children (6, 15–22). For example, Dugger et al. revealed a significant increase in PA, reduction in SB, and better sleep pattern (earlier and less variability in sleep) among 180 children (aged 7.9 years) who attended summer camp in the US (16). Similarly, Brazebdake et al. (20) reported that more than 73% of summer camp attenders achieved the recommended PA guideline (i.e., 60 min of moderate-to-vigorous PA [MVPA] per day) (23), which was profoundly higher than the international prevalence of active children (24). While previous studies have provided important implications, they have only examined the immediate effects of summer camp; moreover, no study has examined whether the beneficial effects of summer camp on movement behaviors remained after the completion of summer camp (i.e., lasting effects). Additionally, most studies have examined the effects of “day camp (leaving each afternoon)” (6, 15–18, 20, 21) rather than “resident camp (staying overnights)” on movement behaviors (19). As a resident camp provides a more structured environment than a day camp owing to its characteristics (19), the beneficial effects on movement behavior might be greater in resident camp.

Therefore, this study examines whether a resident summer camp could improve children's movement behaviors when they were in camp (i.e., immediate effect). Additionally, we aimed to examine whether the effects (if any) remained once the camp was over (i.e., lasting effect) among Japanese children aged 9–14 years. Here, using repeated pre-, during-, and post-intervention measurements, we examined how children's movement behaviors could change in different contexts (i.e., school term vs. summer camp vs. summer vacation; weekdays and the weekend). Based on SDH, we proposed the following hypotheses.

### Hypothesis 1

*The Resident Summer Camp Improves the Children*'*s Movement Behaviors*.

### Hypothesis 2

*Children engage in unfavorable behaviors (reduction in PA, increased in SB, and later sleep time) during the summer vacation after the camp*.

### Hypothesis 3

*Children engage in favorable behaviors (increases in PA, reductions in SB, and earlier sleep timing) when they are in the school term after the summer vacation*.

### Hypothesis 4

*Children engage in unfavorable behaviors during the weekend compared with weekdays*.

### Hypothesis 5

*The summer camp is effective in minimizing the risk of weight gain*.

### Hypothesis 6

*There is no lasting effect of the summer camp on the children*'*s movement behavior*.

## Materials and Methods

### Study Design

**Figure 1:**
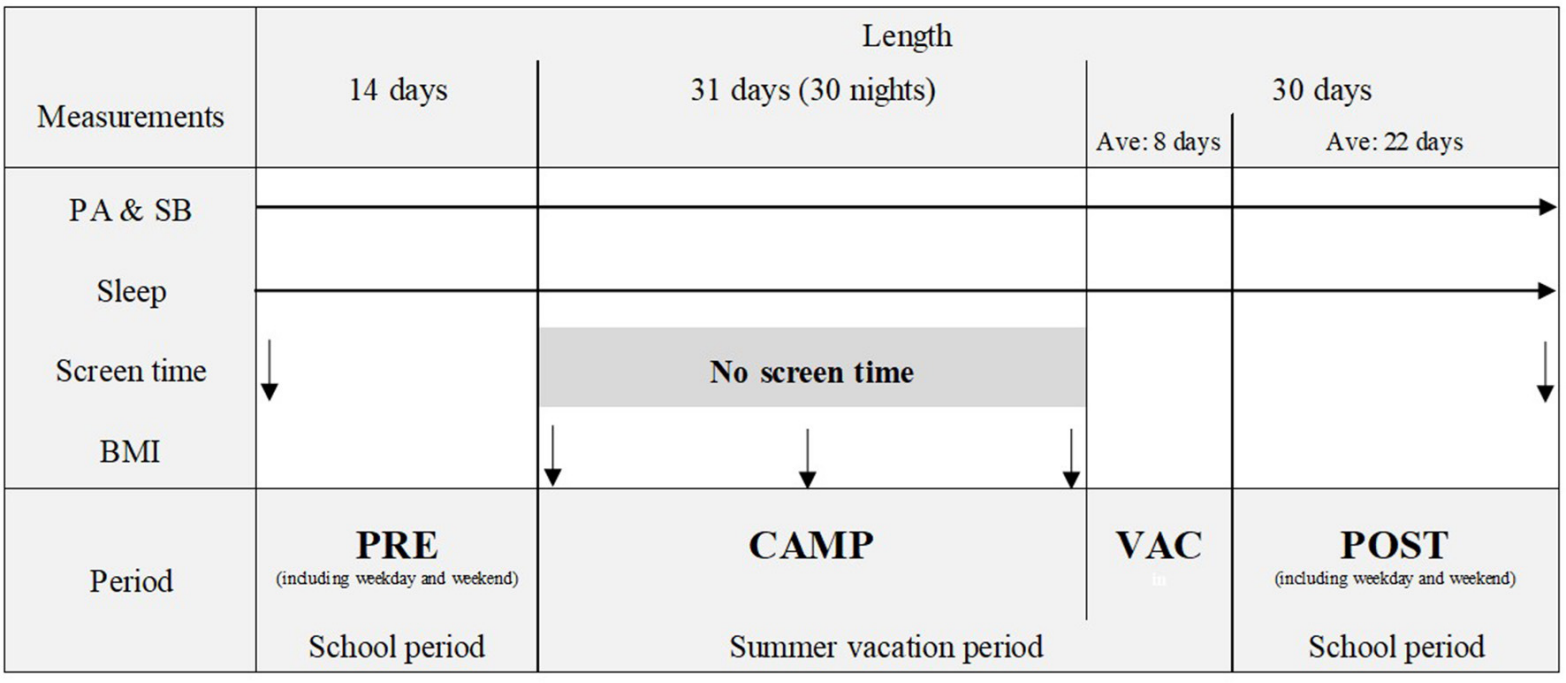
Study overview. PA, physical activity; SB, sedentary behavior; BMI, body mass index. PRE, prior to the summer camp (date: between July 9 and July 22, 2021); CAMP, during the summer camp (date: between July 23 and August 22, 2021); VAC, during the summer vacation after the camp (date: between August 23 and the start of the new semester); POST, during the new semester after the summer vacation (date: between the start of the new semester and September 21, 2021). ↓, Measurements for screen time or BMI. → , Daily measurements for PA, SB, and sleep for the entire study period.

In total, 21 children participated in a repeated within-subject study. The study period included a 31-day resident summer camp (CAMP) with pre- (PRE: 14 days) and post- (30 days follow-up) assessments of the camp. As there was a time gap between the end of the summer camp and the start of the new semester at schools, the post assessment included two periods (i.e., during summer vacation: VAC and during school period: POST). Additionally, as the first day of the new semester differs depending on schools, the lengths of the periods varied across participants (average days for VAC and POST were 8 days and 22 days, respectively). In the end, the study period was divided into the following four periods: PRE vs. CAMP vs. VAC vs. POST. During the study period, the PA, SB, and sleep were continuously measured daily for the entire study period (75 days; between July 9 and September 21, 2021). Recreational screen time was measured in PRE and POST. Furthermore, the body mass index (BMI) was measured three times during CAMP. The study was conducted in accordance with the Declaration of Helsinki and approved by the institutional ethical committee of Nippon Sport Science University (Approval No. 021-H064). The study overview has been presented in Figure 1.

### Resident Summer Camp

The nature-based residential summer camp (31 days) was established by Hello Woods, Twinring Motegi (Honda Mobilityland, Co., Ltd., Mie, Japan) in 2002; it has been held during summer vacation every year ever since. The camp includes multiple educational (e.g., health-related lectures), outdoor (e.g., learning survival skills such as using a knife and lighting a fire), and sports activities (e.g., riding motor bikes and canoeing). The children's time was mostly filled with activities pre-planned by the organizations. On a typical day, the children woke up around 4:30 a.m. and went to bed around 8:00 p.m. After waking up in the morning, the children ran 3 kms in the trail running course. Thereafter, they engaged in a series of pre-planned morning and afternoon sessions along with cooking, eating three meals, and taking a bath. Except for the everyday routine activities (i.e., cooking and eating three meals, morning trail running, washing clothes, taking a bath, and sleeping), the children performed a series of pre-planned educational, outdoor, and sports programs, which differed each day (on an average, the duration of the programs each day was around 6 h). They were not permitted to bring or use any electronic devices; therefore, their screen time during the camp was none. The summer camp program was open to children aged 9–15 years across Japan who paid to participate in the program. The camp was held between July 23 and August 22, 2021. In total, 21 children aged 9–14 years enrolled in the summer camp program. Among them, all parents or guardians provided a written informed consent for their children's participation in the study. Therefore, the final sample size of this study was 21 children (17 boys and 4 girls; 10.3 ± 1.2 years). The participants' characteristics at the pre-assessment of the camp are shown in Table 1.

**Table 1:** Participants' descriptive characteristics at pre-assessments of the camp.

***n =* 21**	**Mean ±SD**
Age (years)	10.3 ± 1.2
% Boys (%)	81.0 (*n =* 17)
Height (cm)	141.8 ± 10.0
Body mass (kg)	36.1 ± 8.6
BMI	17.7 ± 2.3

*BMI, body mass index*.

### Measurements

#### Physical Activity and Sedentary Behavior

The PA and SB were assessed by using the three-axis accelerometers (ActiGraph wGT3X-BT, LLC, Pensacola, FL). The accelerometers have been shown to be valid and reliable activity monitors for measuring PA and SB in children (25, 26). The participants were asked to wear the accelerometer on the right side of their hip using a belt except while sleeping or during water-based activities (e.g., showering or swimming). We asked them to wear the accelerometers during the entire study period (between July 9 and September 21, 2021). Data were collected in 15 s epochs. Non-wear time was defined as a period of ≥ 60 min of continuous zero counts as recorded on the ActiGraph (27). Only the days with ≥10 h of wear time for a minimum of 4 days (including at least 1 weekend day) from the four periods (PRE, CAMP, VAC, POST) were included in the analyses (28). Evenson's cut-off points (26) were used to categorize the activities into three levels: SB, <101 counts per min (CPM); light-intensity PA (LPA), 101–2295 CPM; moderate-intensity PA (MPA), 2296–4011 CPM; vigorous-intensity PA (VPA), ≥4012 CPM; MVPA, ≥2296 CPM. The percentage of SB, LPA, and MVPA was calculated by dividing the mean minutes for each intensity by the total wear time multiplied by 100 (29, 30). The collected data were analyzed using the ActiLife software, version 6.13.3 (ActiGraph, LLC, Pensacola, FL). Weekday and weekend PA and SB were calculated by averaging the weekday (Monday–Friday) and weekend (Saturday, Sunday, and holiday) PA and SB, respectively.

#### Recreational Screen Time

Recreational screen time was assessed using a questionnaire based on national surveillance (31). It was measured at two points: PRE and POST. The participants were asked the following six questions about their screen usage. (1) On average, for how long do you watch TV, videos, and DVDs per day? (2) On average, for how long do you play electronic games per day (such as Nintendo Switch and PlayStation)? (3) On average, for how long do you use a smartphone per day? (4) On average, for how long do you use a mobile phone per day? (5) On average, for how long do you use a PC per day? (6) On average, for how long do you use a tablet per day? Participants were asked to report the time spent on each screen behavior for weekdays and the weekend, respectively. Thereafter, the weekday and weekend screen times were calculated by summing up the six screen behaviors. Weekly screen time was estimated by taking a weighted average of the daily weekday and weekend screen time (i.e., weekly screen time = ([weekday screen time × 5] + [weekend screen time × 2])/7).

#### Sleep

Sleep behavior was assessed by self-report using a daily log. The participants were asked to report their bed or wake time during the whole study period (between July 9 and September 21, 2021) and accordingly, the sleep duration was calculated daily. We calculated the midpoint of sleep (defined as the middle time point between bedtime and waketime) because the midpoint of sleep was reported to be significantly associated with higher risks of cardiovascular disease, obesity, and depressive symptoms (32). Weekday and weekend sleep durations were calculated by averaging the weekday (Monday to Friday) and weekend (Saturday, Sunday, and holiday) sleep, respectively.

#### Anthropometry Assessment

Anthropometry assessments were conducted thrice during the summer camp; T1 (day 2; July 24, 2021), T2 (day 15; August 6, 2021), and T3 (day 30; August 21, 2021). Body mass was measured using a digital weighing scale accurate to the nearest 0.1 kg, and height was measured using a tape measure accurate to the nearest 0.1 cm. BMI was calculated as body mass (kg) divided by height in meters squared (m^2^).

### Statistical Analyses

Mixed models for repeated measures were performed to examine the changes in PA, SB, sleep, screen time, and BMI during the study period. If the *p*-value from the mixed models was less than the significance level, *post hoc* tests were performed. All statistical analyses were performed using IBM SPSS Statistics for Windows, version 27.0 (IBM Corporation, Armonk, NY, USA), and a *p*-value of <0.05 denoted statistical significance. Values were reported as means ± standard deviation unless otherwise stated.

## Results

### The Effects of Resident Summer Camp by Hypotheses

Hypothesis 1: *The resident summer camp improves the children's movement behaviors*.

Table 2 shows the changes in PA, SB, and sleep for before and during the camp (PRE vs. CAMP). All the participants provided valid data; therefore, the final sample size was 21 children (17 boys and 4 girls; 10.3 ± 1.2 years). Consistent with Hypothesis 1, the children engaged more time in MVPA and LPA, less time in SB, and had more step counts in CAMP compared with PRE. Additionally, they exhibited earlier bed or wake time and midpoint of sleep time in CAMP compared with PRE. There was no significant difference in the sleep duration between PRE and CAMP (Table 2). Furthermore, as the participants were not allowed to use any electronic devices during the camp, their screen time during the camp was nil compared with 144 min per day in recreational screen time in PRE.

**Table 2:** Changes in physical activity, sedentary behavior, recreational screen time, and sleep behaviors during the study periods.

		**PRE**	**CAMP**	**VAC**	**POST**	***p*-values**	**Comparisons**
Physical activity	SB (%)	66.1 ± 5.1	58.0 ± 7.1	67.3 ± 4.8	68.3 ± 6.5	*p < * 0.001	**CAMP** < PRE, VAC, POST
	LPA (%)	29.0 ± 4.5	32.4 ± 5.8	27.9 ± 4.3	25.8 ± 5.5	*p < * 0.001	PRE, VAC, POST < **CAMP**
	MVPA (%)	5.0 ± 1.3	9.6 ± 1.9	3.6 ± 1.2	4.3 ± 1.5	*p < * 0.001	PRE, VAC, POST < **CAMP**
	Step counts (steps/day)	8,658 ± 1,539	22,405 ± 3,265	7,719 ± 1,614	8,678 ± 1,887	*p < * 0.001	PRE, VAC, POST < **CAMP**
	Accelerometer wear time (min/day)	840.5 ± 71.4	980.0 ± 86.5	829.1 ± 58.5	817.2 ± 73.4	*p < * 0.001	PRE, VAC, POST < **CAMP**
Sleep	Bed time (time)	21:35 ± 0:51	20:08 ± 0:09	20:58 ± 0:41	21:12 ± 0:53	*p < * 0.001	**CAMP** < PRE, VAC, POST
	Wake time (time)	6:20 ± 0:45	4:41 ± 0:12	6:06 ± 0:54	6:17 ± 0:38	*p < * 0.001	**CAMP** < PRE, VAC, POST
	Midpoint of sleep (time)	1:58 ± 0:42	0:24 ± 0:41	1:32 ± 0:41	1:44 ± 0:40	*p < * 0.001	**CAMP** < PRE, VAC, POST
	Sleep duration (h/day)	8.4 ± 0.4	8.3 ± 0.2	9.1 ± 0.4	9.1 ± 0.4	*p =* 0.008	**CAMP** < VAC, POST
Recreational screen time (min/day)	143.6 ± 88.4	**No screen**	-	154.5 ± 98.4	*p =* 0.561	N.S.

*SB, sedentary behavior; LPA, light-intensity physical activity; MVPA, moderate-to-vigorous physical activity; PRE, prior to the summer camp; CAMP, during the summer camp; VAC, during the summer vacation after the camp; POST, during the new semester after the summer vacation*.

*Bold values represent statistically significant p-value (<0.05) between CAMP and the other periods (PRE, VAC, and POST)*.

Hypothesis 2: *Children engage in unfavorable behaviors (reduction in PA, increase in SB, and later sleep time) during the summer vacation after the camp*.

Table 2 shows the changes in PA, SB, and sleep for during and after the camp (CAMP vs. VAC). Consistent with Hypothesis 2, the children engaged less time in MVPA and LPA, more time in SB, and had fewer step counts in VAC compared with CAMP. Additionally, later bed or wake time and midpoint of sleep time in VAC compared with CAMP was noted. In contrast, the children slept for a longer time in VAC compared with CAMP (Table 2).

Hypothesis 3: *Children engage in favorable behaviors (increases in PA, reductions in SB, and earlier sleep timing) when they are in the school term after the summer vacation*.

Table 2 shows the changes in PA, SB, and sleep for during and after the camp (VAC vs. POST). Inconsistent with Hypothesis 3, there were no significant differences in PA, SB, and sleep between VAC and POST (Table 2).

Hypothesis 4: *Children engage in unfavorable behaviors during the weekend compared to weekdays*.

**Figure 2:**
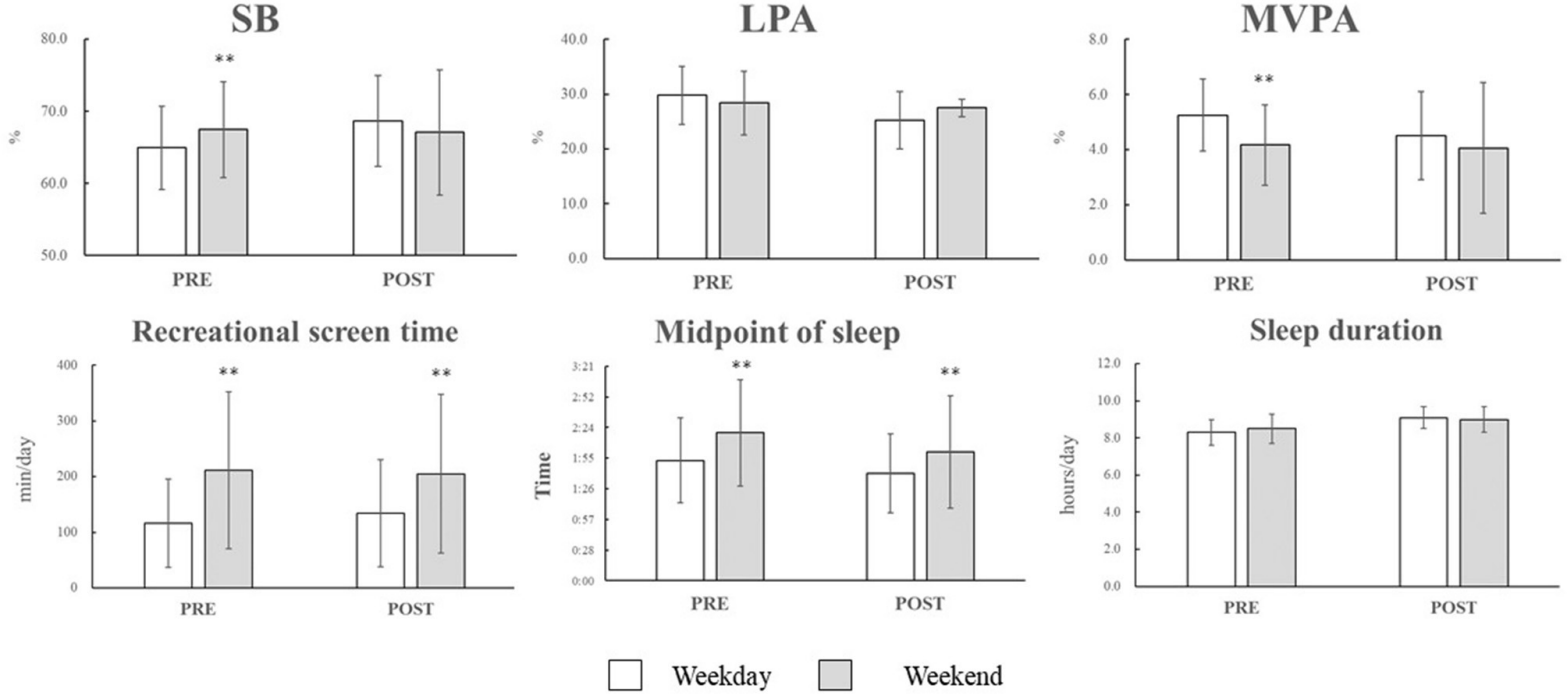
Differences in movement behaviors between weekday and weekend for PRE and POST periods. SB, sedentary behavior; LPA, light-intensity physical activity; MVPA, moderate-to-vigorous physical activity; PRE, prior to the summer camp; POST, during the new semester after the summer vacation. (*n* = 21). At PRE, accelerometer wear time (minutes per day) for weekday and weekend were 861.1 ± 61.8 and 795.3 ± 101.5, respectively. At POST, accelerometer wear time (minutes per day) for weekday and weekend were 824.1 ± 79.5 and 794.1 ± 81.3, respectively. **Significantly different from weekdays (*p* < 0.05).

Figure 2 shows the differences in movement behaviors between weekdays and the weekend for PRE and POST periods. Consistent with Hypothesis 4, the children spent more time in recreational screen time on the weekend than on weekdays in both PRE and POST. Additionally, children's sleep timing shifted to later on the weekend than on weekdays in both PRE and POST (consistent with Hypothesis 4). For PA and SB, the children spent more time in SB and less in MVPA on the weekend than on weekdays in PRE (consistent with Hypothesis 4). However, there was no significant difference in PA and SB between weekdays and the weekend in POST, which is inconsistent with Hypothesis 4 (Figure 2).

Hypothesis 5: *The summer camp is effective in minimizing the risk of weight gain*.

Consistent with Hypothesis 5, there were no significant changes in BMI during the summer camp (PRE =17.7 ± 2.3, Middle = 17.5 ± 2.2, POST = 17.6 ± 2.2, *p* = 0.554).

Hypothesis 6: *There is no lasting effect of the summer camp on the children's movement behavior*.

Table 2 shows the changes in PA, SB, and sleep for before and after the camp (PRE vs. POST). Consistent with Hypothesis 6, there was no significant difference in PA, SB, and sleep between PRE and POST. Additionally, there was no significant difference in recreational screen time between PRE and POST as well (Table 2).

## Discussion

This study examined the effects of resident summer camp on movement behaviors among children using a theoretical framework, the SDH (7). Overall, our findings were consistent with SDH suggesting that children engage in less favorable behaviors in less-structured environments (e.g., weekends and summer vacation) compared with more-structured environments (e.g., weekdays, summer camp, and school term). Indeed, our findings suggest that children spent more time in MVPA and LPA, less time in SB, and had earlier sleep timings (earlier bed/wake and midpoint of sleep time) during the summer camp. However, children's movement behaviors were significantly deteriorated when they were in summer vacation after the camp. Additionally, no significant lasting effects of the summer camp on movement behaviors during the post-intervention period were found. These results suggest that resident summer camp is effective in improving children's movement behaviors when they are in the camp; however, the lasting effects are negligible or at least limited when the camp is completed.

### Immediate Effects of Summer Camp on Movement Behaviors

Our findings show significantly higher MVPA (9.6%), more step counts (22,405 steps per day), and less SB (58.0%) during CAMP compared with the other periods (PRE, VAC and POST). PA levels during the summer camp were higher compared with previous studies reporting PA levels among children attending “day camp” (7.5% for MVPA, 66.8% for SB, and 11,916 steps per day) (15, 19) and were almost equivalent with a result from a previous study reporting step counts among resident campers (19,699 steps per day) (19). Importantly, the summer camp covered most of the summer vacation (on average, 79.5% of coverage) among the participants. Therefore, the camp helped the children in maintaining healthy behaviors during summer vacation. Indeed, in this study, the children's BMI was maintained during the summer vacation when accelerated weight gain was reported by previous studies (1–4). In addition, significant reductions in MVPA (from 10.3–10.6 min/day) and step counts (from 1,825 to 2,292 steps/day) were reported during the summer vacation compared with the school term among Japanese children who do not attend summer camp (11). These results suggest that participation in the summer camp improved children's movement behaviors, which helped to maintain body weight during the summer vacation among camp participants.

### Lasting Effects of Summer Camp on Movement Behaviors

While the summer camp successfully improved children's movement behaviors, these beneficial effects seemed to vanish after camp completion. It was also consistent with SDH, suggesting that children engage in unfavorable behaviors in less-structured environments (e.g., summer vacation) (7). This was also consistent with a previous review study suggesting that the effects of PA intervention were diminished or attenuated when the intervention was completed (33). Additionally, although the children managed to live without any electronic devices during the camp (no screen time for 31 days), the time spent in using recreational screens demonstrated a bounce back to the baseline value in POST. These results may imply that children's behaviors are greatly influenced by the surrounding environments which can be explained by the ecological model (34, 35). Therefore, future studies should consider social (e.g., parental influences) and physical environments (e.g., the availability of electronic devices in households) to induce the long-lasting beneficial effects of summer camp on children's movement behaviors.

### Changes in Weekend PA Behavior After the Camp

Although the lasting effects of summer camp were limited, this study found changes in weekend PA behaviors in POST compared with PRE. More specifically, although the children engaged less time in MVPA and more time in SB during weekends prior to the summer camp (PRE), there was no significant difference in MVPA and SB between weekdays and the weekend after the camp (POST). Previous studies predominantly reported that children engage in less MVPA and more SB during the weekend than on weekdays (7, 36–38). However, it was also reported that weekend behaviors greatly varied across individuals, and some children engage in more MVPA during weekends than on weekdays (39). This is because weekend behaviors are more likely to be influenced by the children's own choices; therefore, some children who preferred PA (e.g., belong to a sports club, high self-efficacy, and a positive attitude to PA) (39–42) could maintain preferable PA behaviors during weekends. In this study, we confirmed that sports club participation did not differ between PRE and POST. Therefore, other individual factors including self-efficacy and attitudes to PA might have changed after the highly active summer camp (on average, 22,405 steps/day for 31 days), which might have caused changes in weekend PA behaviors among the children. However, we did not collect any data to support this hypothesis; therefore, future studies should also consider those individual factors that are related to PA.

### Strengths and Limitations

This study has several strengths. With the post-intervention measurements, this study was the first to examine the immediate and lasting effects of resident summer camp on movement behaviors on children. The repeated data collections enabled us to understand how children's movement behaviors were changed in different contexts (i.e., school term vs. summer camp vs. summer vacation; weekday and weekend). Additionally, using accelerometers and the daily log, we continuously monitored participants' PA, SB, and sleep data for the entire study period (75 continuous days). Continuous monitoring is important to accurately capture children's movement behaviors as these behaviors are known to vary on a day-to-day basis (43). Furthermore, this study used the theoretical framework (SDH) to propose research questions and interpret the results, which are widely considered as a best practice in research (44).

Despite the insights provided in our study, some limitations need to be addressed. First, this study did not include a control group (i.e., non-camp attenders); therefore, the certainty about our results was limited. Indeed, seasonal variations in movement behaviors have been previously reported (11, 45); however, our continuous monitoring may mitigate the risk of distorted results from the seasonal variation. Second, we included only few children from one resident summer camp; therefore, the generalizability of the results was limited. The effects of the summer camp may differ according to the participants' demographic (e.g., age, sex, and socioeconomic status). Therefore, a larger sample from various summer camp programs is required to address this limitation. Third, we did not measure any food intake data; therefore, it might be possible that the balanced food intake during the camp was the other potential reason for the reduced risk of obesity.

## Conclusion

This study showed that the resident summer camp was effective in improving children's movement behaviors while they were in the camp. However, the lasting effects were negligible or at least limited when the camp was completed. Further research is needed to identify maintenance of long-lasting effects of resident summer camp on movement behaviors among children.

## Data Availability Statement

The raw data supporting the conclusions of this article will be made available by the authors, without undue reservation.

## Ethics Statement

The studies involving human participants were reviewed and approved by the Institutional Ethical Committee of Nippon Sport Science University (Approval No. 021-H064). Written informed consent to participate in this study was provided by the participants' legal guardian/next of kin.

## Author Contributions

TK conceptualized the original idea, constructed the methodology, and wrote the original manuscript. All authors have read and approved the final version of the manuscript.

## Funding

This research was supported by the Base Brain Work FY2021 Grant.

## Conflict of Interest

The summer camp was hosted by Hello Woods, Twinring Motegi (Honda Mobilityland, Co., Ltd., Mie, Japan). The company played no role in the study design, analysis or in the preparation of this manuscript. The authors declare that the research was conducted in the absence of any commercial or financial relationships that could be construed as a potential conflict of interest.

## Publisher's Note

All claims expressed in this article are solely those of the authors and do not necessarily represent those of their affiliated organizations, or those of the publisher, the editors and the reviewers. Any product that may be evaluated in this article, or claim that may be made by its manufacturer, is not guaranteed or endorsed by the publisher.
